# Temporal structure of consciousness and minimal self in schizophrenia

**DOI:** 10.3389/fpsyg.2014.01175

**Published:** 2014-10-21

**Authors:** Brice Martin, Marc Wittmann, Nicolas Franck, Michel Cermolacce, Fabrice Berna, Anne Giersch

**Affiliations:** ^1^Centre Référent Lyonnais en Réhabilitation et en Remédiation Cognitive - Service Universitaire de Réhabilitation, Hôpital du Vinatier, Université Lyon 1 and UMR 5229 (Centre National de la Recherche Scientifique), Lyon, France; ^2^Institute for Frontier Areas of Psychology and Mental Health, Department of Empirical and Analytical Psychophysics, Freiburg, Germany; ^3^Département Universitaire de Psychiatrie, Centre Hospitalier Universitaire Sainte Marguerite and Aix-Marseille Université, Marseille, France; ^4^Unité de Neurophysiologie, Psychophysiologie et Neurophénoménologie, UF 4817, Centre Hospitalier Universitaire Sainte Marguerite, Marseille, France; ^5^Laboratoire de Neurosciences Cognitives, UMR CNRS 7291 and Aix-Marseille Université, Fédération 3C, Marseille, France; ^6^INSERM U1114, Department of Psychiatry, Fédération de Médecine Translationnelle de Strasbourg, University Hospital of Strasbourg, University of Strasbourg, Strasbourg, France

**Keywords:** schizophrenia, time perception, self-concept, consciousness, psychology of the self, psychopathology, experimental psychology

## Abstract

The concept of the minimal self refers to the consciousness of oneself as an immediate subject of experience. According to recent studies, disturbances of the minimal self may be a core feature of schizophrenia. They are emphasized in classical psychiatry literature and in phenomenological work. Impaired minimal self-experience may be defined as a distortion of one’s first-person experiential perspective as, for example, an “altered presence” during which the sense of the experienced self (“mineness”) is subtly affected, or “altered sense of demarcation,” i.e., a difficulty discriminating the self from the non-self. Little is known, however, about the cognitive basis of these disturbances. In fact, recent work indicates that disorders of the self are not correlated with cognitive impairments commonly found in schizophrenia such as working-memory and attention disorders. In addition, a major difficulty with exploring the minimal self experimentally lies in its definition as being non-self-reflexive, and distinct from the verbalized, explicit awareness of an “I.” In this paper, we shall discuss the possibility that disturbances of the minimal self observed in patients with schizophrenia are related to alterations in time processing. We shall review the literature on schizophrenia and time processing that lends support to this possibility. In particular we shall discuss the involvement of temporal integration windows on different time scales (implicit time processing) as well as duration perception disturbances (explicit time processing) in disorders of the minimal self. We argue that a better understanding of the relationship between time and the minimal self as well of issues of embodiment require research that looks more specifically at implicit time processing. Some methodological issues will be discussed.

## DISTURBANCES OF MINIMAL SELF AND SCHIZOPHRENIA

There is a consensus that the self is disordered in patients with schizophrenia (for review, Lysaker and Lysaker, 2010; Mishara et al., 2014). Several studies suggest these disorders include a disturbance of the most elementary component of self, i.e., minimal self-disorders (SDs; Gallese and Ferri, 2013; Nelson et al., 2013; Hur et al., 2014). However, characterizing these disorders and understanding the mechanisms involved remain a challenge. In the present work, we bring together ideas and concepts from two different domains, phenomenology and experimental psychology. Combining these two fields is not straightforward, but in the present case such an integrative approach helps us to see the recent phenomenology and timing literature in a new perspective and to discuss the possible impact of timing characteristics on the self. Previously (e.g., Mishara, 2007) it has been proposed there may be a relationship between timing disorders and SDs, but recent data in this field may help to shed new light on this possible link.

### THE MINIMAL SELF

From the point of view of phenomenology, the minimal self is the most basic level of the self. Gallagher (2000, p. 15) defines the minimal self as “a consciousness of oneself as an immediate subject of experience” and as “the pre-reflexive point of origin for action, experience and thought.”

Thus, the minimal self refers to the tacit and pre-reflexive selfhood (Nelson et al., 2013). As explained by Stanghellini (2009) “I experience myself as the perspectival origin of my experiences (i.e., perceptions or emotions), actions and thoughts.” Consequently, the minimal self can be seen separately from more elaborate aspects of the self (Gallagher, 2000; Parnas and Handest, 2003; Nelson et al., 2013) such as the reflexive self (e.g., the explicit awareness of an “I”) and the narrative self (e.g., experiencing the self as having special characteristics, like a personality, and a personal history that we tell about ourselves; Haug et al., 2012).

It is especially important to distinguish between the reflexive and pre-reflexive self. We base our approach on earlier phenomenological work (de Warren, 2009). Brentano and Husserl in particular distinguish between the object of a perception and the conscious perception itself. de Warren provides the following example as inspired by Brentano and Husserl: “when looking at this tree in my backyard, my consciousness is directed toward the tree and not toward my own act of perception. I am, however, aware of myself as perceiving this tree, yet this self-awareness (or self-consciousness) is not itself thematic”, e.g. not reflexive. (de Warren, 2009, p. 19). According to Brentano, when an object of the outer world is present in our mind we simultaneously apprehend the mental phenomenon itself, but this does not lead to explicit reflection: “every act of consciousness is also implicitly conscious of itself, but only to the extent that it is a consciousness of something other than itself” (de Warren, 2009, p. 77). Put another way, when we say “I perceive a tree,” we are usually focusing on the object of perception, i.e., the tree. The “I,” although not unconscious, is only present implicitly. This self-awareness does not compete with the representation of the tree as long as it remains implicit. It is part of the act of consciousness. As we have already seen above, this pre-reflexive “mineness” of conscious experience is a central characteristic of the minimal self.

In contrast, a reflexive act of consciousness is described as reflexive when intentionality is directed toward the act of consciousness itself. As regards de Warren’s example of the tree, “I can, through a further act of reflexion, make my perceptual act into theme, or object, of my consciousness, in which case I am no longer immersed in my directedness towards the tree, but redirected toward myself as perceiving the tree” (de Warren, 2009, p. 20). In this case, self-consciousness becomes reflexive, i.e., an explicit theme through an act of self-observation, which is distinct from a pre-reflexive perspective. Contrary to the minimal self, the explicit representation of self competes with other representations. We either perceive the tree or think about ourselves.

Although these distinctions have important limitations (see Zahavi, 1999), they will be sufficient for the minimal SD issues addressed in this paper. In particular, philosophers have struggled with the Brentano’s proposal according to which the act of consciousness may entail different objects simultaneously: “the presentation of the sound and the presentation of the presentation of the sound (i.e., hearing) form a single mental phenomenon; it is only by considering it in its relation to two different objects, one of which is a physical phenomenon and the other a mental phenomenon, that we divide it conceptually into two presentations” (de Warren, 2009, pp. 78–79). Here, we interpret this proposal like de Warren by considering self-awareness to be present during an act of perception without being the focus of attention, i.e., in an implicit way. It is clear that the concept of self as we discuss it here is not the purest and most abstract form, which would be independent of time and would precede our interaction with the outer world (see Mishara, 2007; Mishara et al., 2014 for a critic of the latter approach), but rather an embodied self. It is during our experience of the world that we implicitly experience ourselves. Consequently, if our experience of the world is distorted, then our implicit experience of ourselves is bound to be disturbed as well. The following clinical examples help further define what we mean by minimal SDs.

### DISTURBANCES OF THE MINIMAL SELF AS A TRAIT MARKER OF SCHIZOPHRENIA

Evidence from empirical research and clinical analysis suggest that a disturbance of the minimal self (minimal-SDs) can be considered a core feature of schizophrenia. Minimal SDs have been described in prodromal, early, and more chronic stages of schizophrenia (Møller and Husby, 2000; Parnas et al., 2003, 2005; Raballo et al., 2011). They are described through a broad range of experiences, as listed in detail in the Exploration of Anomalous Self Experience (EASE), a phenomenology-oriented instrument centered on the exploration of minimal SDs (Parnas et al., 2005). This EASE sets out to list recurrent experiences of a distortion of one’s first-person experiential perspective. The following examples (translated from French by Brice Martin) illustrate patients’ responses (Parnas et al., 2005):

*Alterations of the ‘stream of consciousness’* can be understood as there being a gap between one’s own thoughts and the self, leading to the loss of “mineness” of mental experience. One example of this is “thought interference,” like, for example, in a patient’s own words *“when I’m thinking, you see, sometimes, it’s a bit like…there are some words…some words or ideas which come into my mind…in a disconnected fashion…, which have nothing to do with what I’m thinking,…which can be banal…, which interfere with what I’m thinking….”*

*The Alteration of the Presence* corresponds to a broad range of phenomena that can be defined as a lack of immersion in the world. An example of the alteration of the presence is “a loss of natural evidence” (Blankenburg, 1971). It denotes a “lack of automatic, pre-reflective grasp of the meaning of everyday events, situations, people and objects” (Parnas et al., 2005) as, in the words of a patient, *“you see, for me, it’s a bit like…as if nothing was obvious for me…The world is something…very complicated for me…It’s tiring because I’m always thinking… I’m constantly wondering how people are going so easily through life, through things… Everything is a question for me…you see, I think about everything, and I can’t help it…my mother always tells me I spend too much time wondering about too many things…for example… yesterday… I sat down in front of a wall, and, for one or two hours, asked myself how it had been built? Sometimes, I wonder why “paper” is called “paper”?”.*

*‘Corporeal experiences’ or ‘disembodiment’* denotes the feeling of being detached from oneself and one’s actions, as if in a third-person perspective or without any perspective at all. One example of this is the “spatialization of bodily experiences” where the body is experienced as an object, with a weakened self experience, as in the following narrative: *“mmh…you see, it’s like my body…it’s like I can perceive inside my body, like things being a bit disconnected from my body…I frequently feel my heart beating…or the blow flowing in my veins…I can feel it, it’s as if I can see it…or my muscles when I’m moving my arm…it’s a bit like my body was constantly…was constantly present you see…as a thing in front of me…as if I wasn’t really inside my body….”* A loss of self thereafter means a disconnectedness that can be described as detachment from one’s physical body (de Haan and Fuchs, 2010).

The *altered sense of demarcation* can be understood as a difficulty discriminating self from not self, and, consequently, as a “loss or permeability of self-world boundary” (Parnas et al., 2005). One illustration of this can be seen in the following patient’s explanation: “*you see, I’m like a house with a door that is constantly open…I’m living the fact that…as if people could come in…as if people could know what I’m thinking… As if… I’ve no barrier…as if there was no barrier between me and others…”*

All the above disturbances can be described as a loss of the center of gravity of experience (the “zero point”).

The consideration of minimal SDs in psychiatry may have considerable applications, especially in differential diagnosis, inasmuch as SDs differ in schizophrenia and in bipolar disorders (Parnas et al., 2003). The evaluation of these minimal SDs may also be relevant for the *outcomes of schizophrenia*, such as suicidal behaviors, which appear to be correlated with the intensity of minimal SDs (Skodlar and Parnas, 2010). To date, however, detection of these disturbances relies on the verbal reports given by the patients. Such reports should be interpreted with caution, especially since the minimal self is related to non-verbal aspects of consciousness. The difficulty is to find a way of objectifying the minimal self with a non-verbal approach, while ensuring that this approach is relevant to the minimal self (Mishara, 2007; Mishara et al., 2014). Below, we discuss the possible role of time processing, which may be one of the key mechanisms underlying certain properties of the minimal self (Kiverstein, 2009). We sum up existing arguments in favor of elementary timing disorders in patients with schizophrenia and argue that such perceptual timing disorders should impact the sense of self, inasmuch as temporal aspects are involved in any states of feeling, whether regarding the self or the outer world.

## COGNITIVE BASIS OF MINIMAL SELF DISTURBANCES IN SCHIZOPHRENIA: TOWARD THE QUESTION OF TIME?

Little is known about the basic processes and cognitive correlates of minimal SDs. The question we ask here is whether known cognitive disturbances in patients with schizophrenia lead to minimal SDs. We present a synthetic review of works targeting this issue.

### CLASSICAL NEUROCOGNITIVE DISTURBANCES IN SCHIZOPHRENIA AND SDs

Neurocognitive impairments appear to be a core feature of schizophrenia, having a major impact on everyday functioning (Green, 1996; Green et al., 2000). The most common disturbances concern attention, memory, and executive processing (Heinrichs and Zakzanis, 1998).

These disturbances are frequently considered to be an important source of difficulties for patients in everyday life. For example, they are commonly considered to be the most important factor underlying the functional disabilities observed in schizophrenia, impinging upon patients’ daily lives (Velligan et al., 2006) and employment. They constitute the target for cognitive remediation, a promising therapeutic tool (Demily and Franck, 2008).

Despite their frequency and importance in terms of disabilities, the link between these cognitive disturbances and minimal SDs in schizophrenia is unclear. Very few studies have attempted to explore these relationships. Haug (Haug et al., 2012) explored cognitive functioning (psychomotor speed, working memory, executive and memory functioning) and correlated cognitive impairments in schizophrenia with minimal SDs as assessed using EASE. However, they found few correlations between cognitive functioning and SDs.

We argue that cognitive functions as explored in the usual neuropsychological batteries may not be the most suitable approach. Below, we present the results of studies that are potentially closer to the question of the minimal self. There have been many studies that have explored the concept of agency. Agency is the feeling of being the agent of an action and has mostly been explored by explicitly asking subjects whether or not they are at the origin of a given action. As such, agency is reflexive rather than pre-reflexive. However, the mechanisms hypothetized as being involved in this emerging feeling are largely unconscious and pre-reflexive and could affect the minimal self. Moreover, some observations raise the question of timing. For these reasons, we start by presenting a summary of the studies exploring agency.

### INTERNAL MODELS AND MINIMAL SDs

The last few decades have seen the emergence of a set of cognitive hypotheses targeting some of the clinical features of schizophrenia such as delusions of alien control (first rank symptoms according to Schneider, 1995) and the delusional feeling of being controlled or influenced by other agents, both common manifestations in schizophrenia. Although these manifestations appear to reflect disorders of the reflexive self, they may still be connected with minimal SDs. Indeed, there are a number of arguments that have led us to develop the question of agency despite its being self-reflexive. In fact, impaired agency is observed mainly in acute phases of schizophrenia and only affects patients permanently in exceptional cases, although some disorders might persist in the interval between acute phases. It has been proposed that impaired agency emerges as a result of a combination of impairments, some of which would not be explicit and might be related to minimal SDs (Frith, 2005). Such impairments would lead to a loss of control, a frequent feeling which can arise, for example, when an action has not been performed in an optimal fashion (Pacherie, 2008). While not resulting in a loss of agency, it may weaken our implicit sense of being at the origin of our action. Insofar as it is not reflexive, such a loss of control might thus be connected with minimal SDs. It is additional impairments, such as abnormal interpretations of causal relationships, which would lead to explicit agency impairments and delusions of control. To make our arguments more concrete, we detail below the mechanisms that have been hypothesized as being involved in the loss of control. This in turn raises time issues.

Empirical work has been conducted in connection with “forward models,” including mechanisms not related to conscious awareness. According to this approach, the sense of agency among healthy subjects is based on mechanisms that allow us to prepare an action and to translate intentions into actions (Frith, 2005). Based on Wolpert’s (1997) model, the intention is translated into a motor program by means of an “inverse” model which allows the motor system to adjust the motor program to the intention even before the movement is initiated. Desmurget and Sirigu (2012) proposed that these first steps are associated with both a “wanting to move” and an “urge to move.” Such feelings may be conscious, but they are not self-reflexive. Like the example given above of perceiving a tree, when we want to move, we focus on the action, and not on the “I” performing the action. It is worth emphasizing that we do not discuss the issue of will in this paper. “Wanting” to move suggests there is a conscious decision prior to moving. However, when we take a conscious decision, we usually focus our attention on the decision itself, not on the “I” taking the decision. Once again, the “I” will only be present implicitly. Yet, insofar as this “I” is defined in relation to the action, impairments in the ability to convert the first steps of action planning into real action might impair the implicit sense of “I,” i.e., the minimal self. In fact, some studies have suggested that some aspects of planning are impaired in schizophrenia in the case of simple action sequences (Zalla et al., 2006; Delevoye-Turrell et al., 2007). For example, it has been shown that patients with schizophrenia have planning difficulties when tapping a surface with their finger. Tapping involves lowering the finger and then lifting it. It has been shown that healthy subjects can plan to lift their finger before the action onset and do not need to wait for sensory feedback. Patients, however, appear to wait for sensory information regarding surface contact before lifting their finger. Similarly, they have repeatedly been shown to be impaired every time separate parts of a movement should succeed each other smoothly (Delevoye-Turrell et al., 2003, 2007). Inasmuch as such impairments may weaken the ability to convert an intention into a controlled action efficiently, it might impact the implicit and pre-reflexive feeling of control associated with motor actions. It is noteworthy that all these impairments are observed when several components of an action must follow one another, and that they are reflected in abnormally long intervals between separate motor elements making up the action.

These findings emphasize the timing component of action in patients (Delevoye-Turrell et al., 2012; Turgeon et al., 2012). This is also the case with another component of motor action, the “efference copy.” Once a motor program is adjusted, it generates an “efference copy,” which is used to predict the sensory outcome of the action by means of the forward model (von Holst and Mittelstaedt, 1950). The action is then adjusted by comparing the expected outcome with the actual sensory feedback occurring as a result of the action. When the expected and actual outcome of the action match, the correspondence of these signals reinforces the sense of having initiated the action (Frith, 2005). It has been proposed that the efference copy is disturbed in patients with schizophrenia (Kelso, 1977; Franck et al., 2001; Jeannerod, 2009; Voss et al., 2010; Synofzik et al., 2013). As a result of this disturbance, patients would not benefit from the match between the predicted and actual outcome of the action. Again, the consequence would be a weakening of the sense of having initiated the action. As already emphasized, this does not lead directly to the delusional belief of being controlled by an external agent, which, in order to develop, would require additional impairments (Frith, 2005). Inasmuch as the weakening of the sense of initiating the action occurs implicitly, without the subject being able to report it explicitly, it might be related to minimal SDs. Interestingly, the mechanisms associated with producing an efference copy might also involve a timing component. The efference copy involves a temporal dimension, inasmuch as it is used to predict sensory feedback, and hence the timing of such feedback. The temporal dimension of the forward model had been underlined by Wolpert himself, and more recently by a series of other authors (e.g., Waters and Jablensky, 2009). Interestingly, the temporal distortion of the sensory feedback appears to impact all patients with schizophrenia and to affect their sense of agency independently of the association with delusions of control (Franck et al., 2001). Time processing disorders might be more stable markers than those ultimately resulting in delusions of control. In other words, timing disorders might be trait markers that persist in chronic phases of the pathology. This is consistent with the idea that such disorders weaken patients, possibly by inducing minimal SDs, but produce agency disturbances only secondarily. Patients may interpret their basic disorders in different ways, which explains why agency disturbances can take many different forms, with patients attributing their action to various agents (e.g., God, extra-terrestrial beings, television). However, these different interpretations would stem from a similar basic impairment. In that sense, the agency studies shed light on minimal SDs in schizophrenia (see Gallese and Ferri, 2013).

Yet, this approach may not provide the full explanation for minimal SDs in schizophrenia. First of all, forward models account for only part of minimal SDs because they target mainly motor action. Consequently, they give preference to exploring the sense of agency based on body perception and do not easily address other clinical manifestations, such as the alteration of presence.

Moreover, some models of the minimal self (Gallagher, 2000) distinguish between two components: the sense of “agency” involving forward models, and the sense of “ownership.” The sense of ownership may be closer to the minimal self than the sense of agency. We feel our body as our own body even when we do not move, or when our action is involuntary rather than deliberate. This feeling corresponds to the sense of “ownership.” As in previous examples, it is not necessarily self-reflexive. We can say “my body” without reflecting on the “my.” The current assumption in the literature is that only one component of the minimal self, the sense of agency, is disturbed in schizophrenia. However, Parnas suggests both aspects of minimal self are concomitantly impaired in schizophrenia. Indeed, the EASE assessment suggests minimal-SDs include both, impairments of ownership and agency (Parnas et al., 2005). However, it is difficult to disentangle ownership and agency disorders, and to the best of our knowledge the implicit sense of ownership has not been extensively explored in patients (but see de Haan and Fuchs, 2010), such that the phenomenological frontiers between these two components of the minimal self need to be more accurately defined. Lastly, it is still difficult to distinguish between the reflexive and pre-reflexive parts of agency, and more work is required in order to understand which aspects of motor impairments relate to minimal SD in schizophrenia and to what extent.

## TIME AND MINIMAL SELF IN SCHIZOPHRENIA: WHAT EVIDENCE IN THE LITERATURE?

After this short review of the motor impairments possibly associated with minimal SDs in schizophrenia, we focus more specifically on exploring the potential implications of time deficits in schizophrenia in minimal SDs.

We shall present two series of arguments to justify time processing as a relevant issue to explore and with a view to understanding minimal SDs in this pathology. Mind structuring disorders, namely arguments deriving from psychopathology and experimental psychology studies in schizophrenia are presented below.

### EVIDENCE STEMMING FROM CLINICAL, PSYCHOPATHOLOGICAL, AND PHENOMENOLOGICAL APPROACHES

From a psychopathology perspective, the phenomenological approach proposes a “useful conceptual framework within which the explanation of pathological experiences could be ventured” (Wiggins et al., 2003), to include the role of time in the genesis of minimal SDs. In what follows we try to summarize the hypotheses put forward with the help of this particular conceptual framework. It should be emphasized that these hypotheses rely not on experimental work but on the understanding of time issues derived from phenomenology and on clinical observations.

A first step has to do with the nature of time processing itself. In phenomenology, time is not necessarily investigated and understood as a content of consciousness. Rather, it is a key component structuring the form of consciousness. Thus, time appears to be a very basic, “ontological” component of reality (Wiggins et al., 2003).

At the lowest layers of world-constituting processes, philosopher Husserl (1991) locates the question of time and, more precisely, what he calls “the intimate consciousness of time” (inneres Zeitbewusstsein). He describes a tripartite structure of time consciousness, that is seen as an integration of the past, the present, and the future. He gives the example of music. When we listen to a tune, we are conscious of the present note but still have the previous note in mind (“retention”) and usually anticipate the note to come (“protention”; Gallagher and Zahavi, 2014). As Fuchs (2007) points out, “these synthetic functions, operating at the most basic layer of consciousness in an implicit, tacit or automatic way, are capable of integrating the sequence of single moments into an intentional arc,” allowing the subject to connect tightly with the world and, thus, structuring consciousness. It should be emphasized that the term “intentional” does not mean the integration of past, present, and future moments is deliberate and reflexive. On the contrary, it is passive. According to phenomenologists, it is a basic mechanism whereby we can consciously experience the world as a whole and as continuous in time. Moreover, such mechanisms would shape all our experiences and affect our sense of self (Fuchs, 2007). The ability passively to integrate past, present, and future moments would allow us to think in meaningful units. In a similar way to what has been described in the domain of motor control, protention would allow us to anticipate the next thought and words when we speak.

This implicit or automatic temporal synthesis (or, in Husserlian terms, the “passive” temporal synthesis) contributes to the stability of the perception of the world. Husserl calls this “doxa,” e.g., the certainty that the world will be invariant, allowing the subject to recognize him- or herself in the world. This is one of the most basic processes guaranteeing that the world can be “taken-for-granted” (Wiggins et al., 2003). “As a consequence, the actions of normal people can presuppose a pre-given world, a spatial, temporal, causal, and social order that their mental lives consistently constitute” in an automatic way (Wiggins et al., 2003).

A considerable number of psychopathological works describe a breakdown of this intentional arc in schizophrenia, which could lead to the first incidents of schizophrenic experiences or, put differently, to minimal SDs (Minkowski, 1933/2005; Binswanger, 1965; Tatossian, 2002) or a “weakened ego” (Wiggins et al., 2003). “If these syntheses should cease to occur in my mental life, the ongoing existence of the world and its objects would cease for me” (Wiggins et al., 2003), and the world can no longer be “taken for granted” (Wiggins et al., 2003). Consequently, because the lowest strata of mental life are impaired, the person feels threatened with a kind of selflessness and worldlessness and experiences “ontological anxiety.” The “selflessness” experienced here is not reflexive. The presence of “selflessness” is hypothesized by phenomenologists who base their argument on how the experience of the world is supposed to lead to the minimal self. Phenomenological inquiry also relies on clinical observations. In particular clinical observations can be reinterpreted as conscious compensation for minimal SDs. Because patients’ experience of the world is distorted, they have to compensate for it. The world has to be actively reconstructed (the person must engage in “rational reconstruction”). A wide range of apparent symptoms can be understood as being a consequence of this fundamental disturbance of minimal SDs, such as hyperreflexivity or the loss of natural evidence. Hence, various aspects of minimal self could be understood or may manifest themselves as an attempt by the patient “to actively busy him- or herself with re-laying the ontological foundations of reality” (Wiggins et al., 2003). This is understandable insofar as there is a balance between the pre-reflexive and reflexive self. If the pre-reflexive self is weakened, patients would offset this weakness by giving explicitly thought to questions we usually ignore (Nelson et al., 2009). Although an interesting idea, it has often been argued, and rightly so, that it is difficult to demonstrate (Mishara, 2007). However, there is also more direct evidence of the distorted world experience of patients, who appear to stray from a lived and dynamic time. A thorough examination of the literature suggests direct experiences of an explicit feeling of time disruption might be more frequent than suggested by clinical experience (Hartocollis, 1983). For example, patients describe feeling that time is standing still. One of Minkowski’s patients gave the following explanation: “I’m looking for the immobility and I have a tendency to immobilize all the things around me” (Minkowski, 1927/2002). Another patient reported: “Things go too quick for my mind. […] It’s as if you were seeing one picture one minute and another picture the next” (Chapman, 1966). Similarly, in the words of another patient, “What is the future? One cannot reach it. […] Time stands still […]. This is boring, stretched time without an end” (Fischer, 1929). In the context of our discussion, the following statement by a schizophrenic patient of Bin Kimura quoted in Fuchs (2013) is the following: “Time is also running strangely. It falls apart and no longer progresses. There arise only innumerable separate now, now, now … quite crazy and without rules or order. It is the same with myself. From moment to moment, various “selves” arise and disappear entirely at random. There is no connection between my present ego and the one before.” As we shall show below, schizophrenia patients’ performance in a specific psychophysical task shows behavior interpretable as a disturbance in the continuity of time, as if they were “stuck” in time and could not progress temporally.

This short excursion into the phenomenological approach suggests time is a key component of experience and, more than a content of consciousness, a dynamic component of consciousness. However, as emphasized above, the evidence is based mainly on conscious introspection and verbal reports, and the conclusions are thus necessarily limited. As we have seen, phenomenological theorization interprets verbal complaints to explain non-verbal, pre-reflexive mechanisms. This could be considered a major flaw in the approach. In the following, we develop the contribution of experimental psychology as regards more hidden aspects of time processing which might usefully complement the phenomenological analyses and patients’ reports.

### EVIDENCE FROM COGNITIVE STUDIES TARGETING THE QUESTION OF TIME

Research into time perception often relies on explicit judgments, meaning that subjects are explicitly asked to make a judgment about a temporal property of external stimuli, such as their duration, order or simultaneity. However, as we have seen above, timing may also intervene incidentally and may be involved in the structuring of consciousness. Indeed, a distinction currently made when exploring time in cognitive science is between “explicit” and “implicit” timing, resting on different neural networks (Coull and Nobre, 2008). As we have seen, explicit processing concerns any mental activity wherever subjects make a deliberate judgment, e.g., about temporal simultaneity or duration. Conversely, implicit processing occurs without a specific instruction and can be either conscious or not (Coull and Nobre, 2008). The implicit processing of time may be especially relevant for minimal self but cannot be explored without considering explicit aspects.

Paradigms based on explicit timing have been used to assess different aspects of temporal processes in schizophrenia. The most typical are “simultaneity judgment” (e.g., assessing the simultaneity vs asynchrony of two stimuli), “temporal order judgment” (e.g., the capacity to order events) and “duration estimation” (e.g., the ability to determine the duration of a time interval; Pöppel, 1997; Wittmann, 1999; van Wassenhove, 2009; Grondin, 2010).

Many studies have been conducted to investigate duration judgment in patients with schizophrenia (Wahl and Sieg, 1980; Tysk, 1983, 1984; Tracy et al., 1998; Volz et al., 2001; Elvevåg et al., 2003; Davalos et al., 2005; Penney et al., 2005; Carroll et al., 2008; Lee et al., 2009; Waters and Jablensky, 2009) or simultaneity judgment (Schwartz et al., 1984; Foucher et al., 2007; Giersch et al., 2009; Schmidt et al., 2011; Martin et al., 2013). The general conclusion of these studies is that patients with schizophrenia find it more difficult than controls to estimate duration (e.g., a greater variability of the performances) and require bigger differences before being able to distinguish the duration of two stimuli. They also require longer inter-stimulus intervals to be able to make temporal order judgments.

However, the significance of these results seems limited, for several reasons. First, there were only a few correlations with clinical dimensions. Moreover, confounding factors such as attention (Brown and Boltz, 2002), memory processing (Zakay and Block, 2004), and emotional characteristics of the stimuli (Droit-Volet et al., 2004; Grommet et al., 2011) may explain the greater variability in performance, especially for duration judgment. Last but not least, the fact that these evaluations rely on explicit judgments implies they explore the content of time and not the temporal structure imposed on consciousness by time processing.

Another way to explore time processing in schizophrenia is to assess “implicit timing.” According to Coull and Nobre (2008) “implicit timing is engaged, even without a specific instruction to time, whenever sensorimotor information is temporally structured.” It appears that this definition of time is similar to the phenomenological description of human time inasmuch as time is seen as an implicit aspect of processing which does not necessarily lead to a content of consciousness but, rather, shapes the conscious experience. Here, we focus on implicit aspects of timing because they might underlie the involvement of timing in minimal self. We first review the evidence that implicit aspects of timing are impaired in schizophrenia and then discuss how this might impact the minimal self.

**FIGURE 1:**
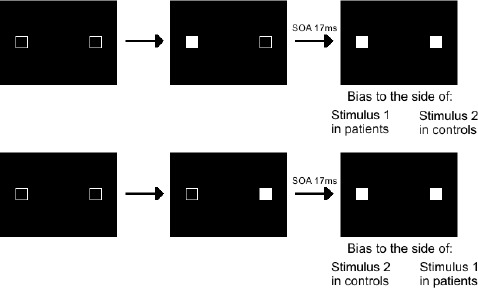
**Illustration of the Simon effect.** For this task, two squares are filled in successively, with an SOA varying between 0 and 92 ms, and subjects are instructed to decide whether the two stimuli are displayed simultaneously or asynchronously. They give their response by pressing a left response key for simultaneous stimuli and a right response key for asynchronous stimuli. Very short SOAs of 17 ms are not detected consciously but yield automatic visuomotor responses representing the processing of events over time. Both patients and controls are biased to respond as a function of the stimulus order, which shows that all subjects distinguish events in time at an implicit level. However, there is a qualitative difference in their bias with the shortest SOAs. Controls are biased to the side of the second stimulus, as if able to follow stimuli in time, whereas patients are biased to the side of the first stimulus, as if stuck with this stimulus and unable to follow stimuli with time. With larger SOAs biases are to the side of the second stimulus in all subjects.

Few studies have explored implicit timing in schizophrenia, particularly in perception. Giersch et al. (2009) used a potentially useful priming paradigm, and Lalanne et al. (2012a,b) used the Simon effect to track temporal processes (Figure 1); Martin et al. (2013) assessed the temporal constraints of multi-sensory integration in schizophrenia; Posada and Franck (2002) explored the automation of rules and Exner et al. (2006) motor sequence learning.

One example of these studies is the one by Lalanne et al. (2012a,b) using the “Simon effect.” In it, subjects have to decide whether two squares are displayed on a screen simultaneously or asynchronously. They respond by hitting a left or right response key (Figure 1). Results revealed an enlarged time window with patients, irrespective of the squares’ position (intra- vs interhemispheric presentation), and independently of a non-specific difficulty with processing the information (Giersch et al., 2009). The implicit processing of asynchrony was explored by means of the Simon effect, which refers to the finding that manual responses are biased (lower reaction times and higher accuracy) to the side of the stimulus independently of the task at hand. Basically, the idea is that when stimuli are presented simultaneously on both sides of the screen, no Simon effect can occur, because information on both sides is perfectly symmetrical. However, when stimuli are asynchronous, there is an asymmetry again between the left and right side, due to the temporal delay. This allowed the authors to measure a Simon effect related to this temporal asynchrony. The results showed that healthy subjects were systematically biased to press the button on the side of the second stimulus (Lalanne et al., 2012a,b), and additional studies suggested they are able to follow stimuli in time at a non-conscious level, i.e., even when they do not consciously detect an asynchrony (Giersch et al., in press). At this non-conscious level, patients also distinguished stimuli in time. However, for asynchronies eliciting “simultaneous” judgments, patients’ responses were biased to the side of the first square instead of the second one.

The authors interpreted these results as evidence of the fact that at an implicit level patients process stimuli as if they are isolated rather than in succession. They propose that this impairment may be related to disturbed predictive coding. Indeed, stimuli which are less than 20 ms apart mean that subjects process a first stimulus while being prepared for another stimulus which may follow. When the second stimulus is presented only 20 ms after the first one, attention cannot be shifted immediately from the first to the second. If after a while the second stimulus is nonetheless processed and prioritized, it means the system is prepared to process a second stimulus and to shift attention toward it. This might be based on a processing loop which allows new events to be anticipated while the focus is still on current information. This loop could be supplied by the recurrent system of information processing described within the predictive coding framework (Friston, 2008), which is in accordance with an embodied approach of predictive interoceptive coding (Seth, 2013). This hypothesis relates the timing results with those observed with motor control, insofar as both outcomes suggest impairments in the predictive loops that allow us to anticipate events, either the consequence of the action or the next perceptual event. The impairment patients have regarding time may reflect a fragmentation of the processing of information that would impact on the sense of time continuity. In particular, it could be linked to the feeling of “frozen time” described above. Indeed, if patients have difficulty following events in time at an implicit level, it would disrupt their ability to process events in a continuous way, matching what some patients explicitly reported. Moreover, it has recently been shown that patients have difficulty discriminating temporal order at a subjective level (Capa et al., 2014), which reinforces the idea that they have difficulty processing the flow of events. Below, we discuss how such impairments could be related to minimal SDs.

### TIME PROCESSING AND THE MINIMAL SELF

If the studies described above doubtlessly suggest impairments at both explicit and implicit levels of time processing (Table 1), their impact on minimal SDs is far from straightforward. Despite the limitations of the phenomenological approach, it provides the concepts that allow us to propose a link between the subjective experience and the minimal self. What the experimental approach provides on top of that is the possibility to objectify the mechanisms subtending the distortion of the world experience. As emphasized at the beginning of this manuscript, and as suggested in some phenomenological approaches, we consider that the basic experience of oneself is intimately associated with the embodied experience of the world, whether during action or perception. It is because we can establish a close link between our intentions and our actions or between what we expect to feel and what really happens that we can implicitly feel we are the one perceiving or acting. Thus, any disruption of our ability to predict or feel the continuity in the flow of our perceptions and actions should result in a disruption of the minimal self. This can apply even in the case of passive movements. There is usually continuity in our perception of our own body, which is confirmed from one moment to the next. If such continuity is disrupted, however, it should affect our implicit feeling of being one.

**Table 1:** **Summary of the results on implicit and explicit timing in schizophrenia.**

Tasks	Main works	Main results in schizophrenia
**Explicit timing**		
Simultaneity judgment	Schwartz et al. (1984); Foucher et al. (2007); Giersch et al. (2009); Lalanne et al. (2012b); Martin et al. (2013)	Patients need larger inter-stimulus intervals than controls to detect asynchrony
Temporal order judgment	Capa et al. (2014)	Altered temporal order judgment (even for asynchronies producing a clear perception of asynchrony)
Duration judgment	Wahl and Sieg (1980); Tysk (1983); Tracy et al. (1998); Volz et al. (2001); Elvevåg et al. (2003); Carroll et al. (2008); Lee et al. (2009); Waters and Jablensky (2009)	Great variability in performance
**Implicit timing**		
Simon effect	Lalanne et al. (2012a,b)	Inability to follow stimuli over short delays
Temporal constraints on multisensory processing	Martin et al. (2013)	Lack of audio–visual integration despite difficulties to detect asynchronies at SOAs > 0 ms
Motor sequence learning and automation of rules	Posada and Franck (2002); Exner et al. (2006)	No benefit from predictability

As emphasized above, the minimal self is a constituent part of the feeling of being present here and now. This implies that both (i) sensory information processing, and (ii) the way information is processed in time play a role in the formation of the minimal self. As already noted, this information does not have to be unconscious to play a role in the minimal self. It only has to exclude an explicit reflection on the self. Diverse information may thus be integrated in the minimal self, including peripheral as well as central sensory information, which conveys information about our body, but also about the environment and how the body behaves in the environment. This shapes our experience and thus yields a sense of self in the environment, prior to language. Besides, being myself “now” also implies having a sense of present time, and since oneself is felt to be continuous in time, it additionally requires a sense of temporal continuity. In other words the minimal self involves many different mechanisms, including timing mechanisms. This means timing impairments should lead to minimal SDs.

We will thus first detail which timing mechanisms are involved in minimal self, and then how deficits in patients with schizophrenia may induce minimal SDs. Let us first consider the sense of present and its possible relation with temporal windows of information processing. The fact that all sensory information is processed continuously from moment to moment necessitates a temporal integration process that binds multimodal information, into one single representation at one present moment^1^ (Pöppel, 1997; van Wassenhove, 2009; Wittmann, 2011). This is the dual nature of time consciousness: the flow of events over time and the feeling of “nowness.” These dual aspects are based on the processing of sequences of events over time and the temporal integration of these successive events through multiple and nested functional brain states of various temporal levels (Northoff, 2013; Fingelkurts and Fingelkurts, 2014). Regarding the relationship between temporal integration windows and the self, however, what is especially crucial is to stabilize this representation for a short duration. Otherwise, our perception would be one of an uninterrupted flow of inputs conveyed by different sensory channels. This would lead to a number of difficulties, and especially a difficulty to integrate these different inputs. When considering only one sensory modality, it has been repeatedly suggested and confirmed that there must be a minimal delay of 20–40 ms between two stimuli for this asynchrony to be detected (von Baer, 1864; Brecher, 1932; Elliott et al., 2006, 2007; Fink et al., 2006; Babkoff and Fostick, 2013). The integration of complex information from different channels within one single representation requires even more time (van Wassenhove et al., 2007). It is, however, not as straightforward as it seems. For example, visual and auditory information travels at different speed, and it must be recalibrated to allow for its integration within a single representation (Pöppel et al., 1990). It is only because the motion of the lips and the syllable utterance are integrated within the same time window and processed as co-temporal that we access the representation of another person speaking instead of disconnected visual and auditory information (van Wassenhove et al., 2007). In a similar way, we integrate sensory information from our body and of the environment in single representations which are stabilized from moment to moment. A disruption of these temporal windows may thus fragilize the minimal self. The lengthening of time windows (Giersch et al., 2009) may represent a mechanism of self-disruption in patients with schizophrenia.

Additionally, and as emphasized above, the representation of ourselves is not only stabilized within temporal windows. The self is generally experienced as being continuous in time. This requires that sensory information be processed and integrated in such a way that we experience continuity over time (Dainton, 2010; Wittmann, 2011; Northoff, 2013). Fragmentation of the mental life would mean a disruption of the continuous processing of the sensory information underlying the minimal self. The implicit processing of information over time might be particularly important for our feeling that we go along with the flow of events in a continuous fashion. Conversely, disruption of these implicit mechanisms in patients may disrupt their feeling of temporal continuity. The fact that this impairment occurs implicitly may also explain why they find it difficult to report this impairment clearly. In summary, a number of time processing impairments might contribute to minimal SDs. First, deficient integration of information within temporal windows may hamper information being stabilized. Second, an inability to process sensory information continuously would lead to fragmentation of the information subtending the minimal self.

Time disorders observed in schizophrenia might thus be closely related to minimal SDs, and might subtend a series of cognitive impairments. The existence of these relationships needs to be confirmed in future studies. Although it is difficult to test minimal SDs directly, it is possible to explore the impact of the impairments described on conscious experience. Such studies involving patients with schizophrenia should provide insight into how time disorders affect our ability to feel ourselves implicitly to be at the center of our perceptions and actions. Thus, the question of whether the alterations observed in the simultaneity/asynchrony judgments are related to the contents of consciousness warrants investigation.

## IMPLICATIONS FOR CURRENT RESEARCH ON TIME AND MINIMAL SELF IN SCHIZOPHRENIA: METHODOLOGICAL CONSIDERATIONS

### NECESSITY OF RELATING PSYCHOPATHOLOGY WITH EXPERIMENTAL APPROACHES

Although there is a growing interest in minimal SDs in schizophrenia, there is an apparent neglect as regards experimental approaches regarding psychopathological hypotheses.

Whereas cognitive research into minimal SDs in schizophrenia rarely includes the issue of time, in the case of cognitive research into time processing in schizophrenia the question of minimal SDs is rarely included. This is all the more paradoxical given that these areas were originally united (Minkowski, 1933/2005), and that psychopathological analysis has gathered data and developed concepts about both for a long time. More recently, however, these two lines of research have developed separately. According to Nelson et al. (2013): “Although progress has been made in understanding phenomenological and neurocognitive disturbances in schizophrenia, these “levels” or domains of enquiry have tended to remain separate from each other”. Combining psychopathological phenomena with experimental approaches seems a promising way to proceed, at least on a heuristic level.

### ASSESSING THE MINIMAL SELF: INCLUDING SUBTLE AND DEMANDING CLINICAL EXPLORATIONS IN RESEARCH PROGRAMS

Assessing minimal SDs requires indisputable clinical expertise, with the help, for example, of EASE, a phenomenologically oriented instrument (Parnas et al., 2005). Regarding EASE, “the interviewer must possess good prior interviewing skills, detailed knowledge of psychopathology in general and of the schizophrenia spectrum conditions in particular.” So “a familiarity with phenomenological description of the structures of human consciousness is indispensable in using the EASE for pragmatic, psychometric purposes” (Parnas et al., 2005).

Consequently, whether using EASE or some other scale, assessing minimal SDs requires that investigators involved in experimental research incorporate subtle clinical analyses within their research programs.

### “TIME OF PERCEPTION” IS NOT “PERCEPTION OF TIME”: ASPECTS OF EMBODIMENT

As claimed in phenomenology, human time is not necessarily a content of consciousness. Accordingly, as stated by Pöppel (2009), “it is important to realize that we cannot perceive time itself.” We believe that paradigms based on implicit time processing as proposed by Giersch (with the Simon effect) or Coull (using automatic temporal accumulation) are promising tasks for exploring correlations between implicit time processing and minimal SDs. However, this does not preclude investigating explicit time, i.e., the judgment of duration, especially since it has been shown that the bodily self is related to time perception in the seconds’ range (Wittmann, 2013). These empirical findings confirm embodied notions of subjective time (Craig, 2009) and link the experiences of emotion, time, and interoception for the creation of a self (Seth, 2013). Inasmuch as the minimal self is related to conscious and embodied experiences, such investigations are necessary for understanding how implicit (and explicit) time processing could affect impairments observed on a more subjective level.

## CONCLUSION

According to research in psychopathology, temporal processing and minimal SDs appear as core components of the schizophrenia condition. In general, the notions of time and the minimal self are two fundamental components of human consciousness. “Time and the self, time and consciousness, affects and time are pairs of subjective reality, phenomena that appear together in the course of man’s ontogenetic development and define human nature […]. Even though conceptualized independently, they cannot be experienced separately, they cannot exist without each other” (Hartocollis, 1983, p. 56). Combining the two dimensions of minimal self and time in experimental studies requires subtle clinical analysis as well as the exploration of implicit time processes, which, by definition, are not reflective. What we have shown, however, is that they can disrupt a patient’s experience of the world at fundamental levels, thus contributing to the minimal SDs. In particular, we presented evidence of our own experimental studies relating to an implicit timing disturbance in patients with schizophrenia which strikingly mirrors patients’ reports of subjective time. We have potentially found an experimental task which directly assesses the implicit timing disturbances of patients with schizophrenia who, on an explicit level, become aware of theses impairments. The underlying mechanisms might also come into play in our ability to feel in control. Experimental approaches can yield further information about the consequences of these basic impairments for how subjects experience their perceptions and actions. We are aware that this is not enough to fill the gap that still exists between the phenomenological approach and experimental investigations exploring elementary aspects of timing, but we argue there is enough ground to at least try to relate minimal self and timing disorders. Furthermore, we propose that exploring the relationships between implicit timing and the temporal structure of consciousness may help further close the gap between the experimental and phenomenological approaches.

## AUTHOR CONTRIBUTIONS

Brice Martin designed and wrote the first draft of the paper, Marc Wittmann and Anne Giersch made substantial contributions to the design of the manuscript, and important revisions. Nicolas Franck contributed to the elaboration of the concepts and the revision of the manuscript regarding clinical aspects. Michel Cermolacce contributed to the elaboration and the revision of the paper regarding phenomenological aspects. Fabrice Berna contributed to the elaboration and revision of the manuscript regarding the concept of self. All authors gave final approval of the submitted version and agreed to be accountable for all aspects of the work.

### Conflict of Interest Statement

The authors declare that the research was conducted in the absence of any commercial or financial relationships that could be construed as a potential conflict of interest.

## Footnotes

1 Although the concept of representation is not at the center of the phenomenological approach, the idea of information integration leads to the notion of representation. In this sense, minimal self would be similar to the concept of core consciousness as defined by Damasio (1998).
